# LncRNA MALAT1 promotes gastric cancer progression via inhibiting autophagic flux and inducing fibroblast activation

**DOI:** 10.1038/s41419-021-03645-4

**Published:** 2021-04-06

**Authors:** Zhenqiang Wang, Xinjing Wang, Tianqi Zhang, Liping Su, Bingya Liu, Zhenggang Zhu, Chen Li

**Affiliations:** 1 Department of General Surgery, Shanghai Key Laboratory of Gastric Neoplasms, Ruijin Hospital, Shanghai Jiao Tong University School of Medicine, Shanghai, 200025 China; 2 Shanghai Institute of Digestive Surgery, Ruijin Hospital, Shanghai Jiao Tong University School of Medicine, Shanghai, 200025 China

**Keywords:** Gastric cancer, Long non-coding RNAs

## Abstract

Autophagy defection contributes to inflammation dysregulation, which plays an important role in gastric cancer (GC) progression. Various studies have demonstrated that long noncoding RNA could function as novel regulators of autophagy. Previously, long noncoding RNA MALAT1 was reported upregulated in GC cells and could positively regulate autophagy in various cancers. Here, we for the first time found that MALAT1 could promote interleukin-6 (IL-6) secretion in GC cells by blocking autophagic flux. Moreover, IL-6 induced by MALAT1 could activate normal to cancer-associated fibroblast conversion. The interaction between GC cells and cancer-associated fibroblasts in the tumour microenvironment could facilitate cancer progression. Mechanistically, MALAT1 overexpression destabilized the PTEN mRNA in GC cells by competitively interacting with the RNA-binding protein ELAVL1 to activate the AKT/mTOR pathway for impairing autophagic flux. As a consequence of autophagy inhibition, SQSTM1 accumulation promotes NF-κB translocation to elevate IL-6 expression. Overall, these results demonstrated that intercellular interaction between GC cells and fibroblasts was mediated by autophagy inhibition caused by increased MALAT1 that promotes GC progression, providing novel prevention and therapeutic strategies for GC.

## Introduction

Inflammatory mediators within the tumour microenvironment (TME) play important roles in promoting gastric cancer (GC) progression. The various cytokines within the GC TME are secreted from inflammatory cells, fibroblasts, and GC cells^1^. Moreover, GC cells could receive extracellular signals, which could further modulate TME via paracrine secretion of cytokine. The cross-talk between GC cells and stroma cells facilitate cancer progression. Cancer-associated fibroblasts (CAFs), a major component of the tumour stroma, are a critical source of various molecules secreted in TME, which stimulate cancer cells progression. Similarly, the fluctuation of inflammatory mediators (growth factors, interleukin) by cancer cells in TME also altered resident fibroblast phenotypes and lead to normal fibroblast (NF) activation, considered as the main CAF source^2,3^. Increasing evidence demonstrated interleukin-6 (IL-6) was abundant in GC TME, facilitating GC progression^4^. Most studies have reported that IL-6 could be released from CAFs and promote GC cells proliferation or metastasis in a paracrine way. However, IL-6 secretion from GC and its effect on modulating TME has not been studied in detail. Here, we have found that autophagy inhibition in GC could upregulate IL-6 expression and secretion.

Autophagy is an important biological process that appears to be a double-edged sword with respect to cytokine signalling and modulating tumour progression in certain instances^5^. Activated autophagy could protect cells from inflammatory damage by inhibiting autophagy and aggravating inflammatory responses in many tissues^5,6^. It is widely accepted that autophagy defects contribute to inflammation and autophagy inhibition under the condition of chronic inflammation devoted to oncogenesis^7,8^. Several reports have suggested that long noncoding RNA (lncRNA) could function as novel autophagy regulators. Silencing lncRNA-FA2H-2 facilitates impairment of oxidized low-density lipoprotein-induced autophagy flux to activate inflammation for increased IL-6 and other cytokine production^9^. Defective autophagy increases inflammatory mediator (such as TNF-α and HGF) production to promote hepatocellular carcinoma^7^. Impaired autophagy could promote chemoresistance in GC via lncRNA ARHGAP5-AS1 accumulation^10^. Although recent studies have shed light on some autophagy impairment mechanisms in GC, the molecular components that mediate the process are yet to be fully identified.

LncRNA metastasis-associated lung adenocarcinoma transcript 1 (MALAT1) has been reported to activate autophagy in pancreatic ductal adenocarcinoma^11^, retinoblastoma^12^, and multiple myeloma^13^ to promote tumour progression. However, the effect of MALAT1 on autophagy in GC has not well reported. In this study, we found that MALAT1 upregulation in GC could inhibit autophagic flux, which led to sequestosome1(SQSTM1) protein accumulation and IL-6 overexpression. SQSTM1 is a scaffold and stress-inducible protein with multiple domains (such as ZZ, LIR, and PBI), which not only acts as an indicator of autophagy flux but also mediates inflammation response^14^. Hence, SQSTM1 protein accumulation might be responsible for IL-6 overexpression in GC cells. The majority of studies have revealed the effect of CAFs on GC cell growth or metastasis within the interaction between CAFs and cancer cells. However, the influence on CAFs exerted by GC cells has not been studied in detail. Autophagy inhibition in cancer cells led to the expansion and release of cytokines. The dysregulated cytokines could activate the transition from NFs to CAFs via paracrine signalling^15^. Here, we found that impairment of autophagy caused by increased MALAT1 could activate NF to CAF conversion through expansion and secretion of IL-6. These data suggest a critical role for MALAT1 in the interaction between CAFs and GCs cells. Furthermore, the underlying mechanisms were investigated to identify potential therapeutic strategies targeting GC.

## Results

### MALAT1 blocks autophagic flux in GC cells

**Fig. 1:**
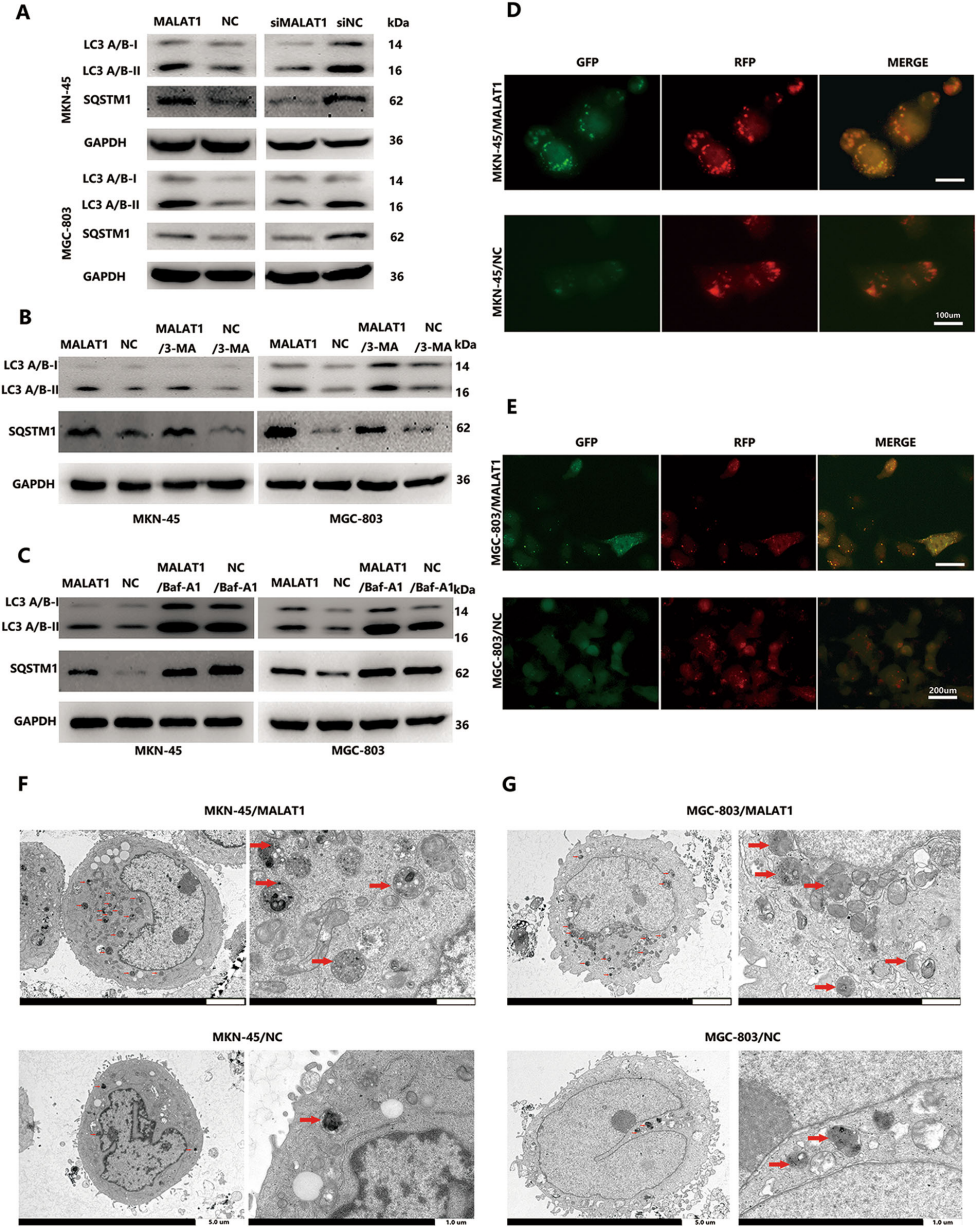
Increased MALAT1 blocks autophagic flux. **A** The LC3 and SQSTM1 protein levels in MKN-45/MALAT1, MGC-803/MALAT1, and their parental cells were determined by western blot assay; **B**, **C** The LC3 and SQSTM1 protein levels in MKN-45/MALAT1 and MGC-803/MALAT1 cells with 3-MA (10 mM) and baf-A1 (10 mM) were determined by western blot assay; **D**, **E** mRFP-GFP-LC3 distribution in MKN-45/MALAT1, MGC-803/MALAT1, and their parental cells were analysed by fluorescence microscopy (MKN-45/MALAT1 vs MKN-45/NC: 50.3 ± 4.5 vs 11.3 ± 2.6; MGC-803/MALAT1 vs MGC-803/NC: 28.3 ± 6.2 vs 2.6 ± 1.6, *P* < 0.01); **F**, **G** The number of autophagic vesicles was increased in MKN-45/MALAT1 and MGC-803/MALAT1 group as seen by TEM.

A previous study documented that MALAT1 could function as an oncogene to promote GC cell proliferation and positively correlated with TNM stages in GC. However, the effect of aberrant MALAT1 expression on autophagic flux in GC was rarely investigated. Anomalous autophagy activity led to a variation of inflammation process^16^. Here, we found that MALAT1 overexpression (Supplementary Fig. 1A) could enhance LC3-1 conversion to LC3-II and SQSTM1 protein accumulation in both MKN-45 and MGC-803 cells. (Fig. 1A, *P* < 0.05). Contrarily, silencing MALAT1 by transducing siMALAT1 (Supplementary Fig. 1B) could inhibit LC3-II and SQSTM1 accumulation (Fig. 1A, *P* < 0.05). Furthermore, MALAT1 had no influence on the expression of SQSTM1 mRNA level (Supplementary Fig. 1C). LC3-II cloud accumulation results from autophagy activation or reduced turnover from autophagosome to autolysosomes. Moreover, the accumulation of SQSTM1 was an indicator of autophagy impairment. Therefore, autophagy inhibitors, 3-methyladenine (3-MA) and bafilomycin A1 (BafA1) were used to treat cells to block autophagy initiation and maturation, respectively. MALAT1 effect on LC3-II and SQSTM1 accumulation were not compromised by 3-MA treatment (Fig. 1B, *P* < 0.05). In contrast, LC3-II and SQSTM1 accumulation was not affected by BafA1 treatment in the MALAT1 overexpression group compared to the negative group (Fig. 1C, *P* < 0.05). Subsequently, an mRFP-GFP-LC3 lentivirus vector was introduced to determine the MALAT1 effect on autophagy flux. When autolysosomes formed, green fluorescence faded, leaving only the RFP signal as the RFP signal is more stable than green fluorescence in acidic conditions. MALAT1 overexpression in MKN-45 and MGC-803 led to yellow puncta enrichment rather than red ones, indicating autolysosome maturation blockage (Fig. 1D, E, *P* < 0.05). In addition, we used TEM to evaluate autophagosomes and found out that the number of autophagic vesicles increased in MKN-45/MALAT1 and MGC-803/MALAT1 cells compared to that in control cells (Fig. 1F, G). Taken together, these results suggested that increased MATLA1 in GC cells could impair autophagy flux.

### MALAT1 activates AKT/mTOR pathway to inhibit autophagy in GC cells

**Fig. 2:**
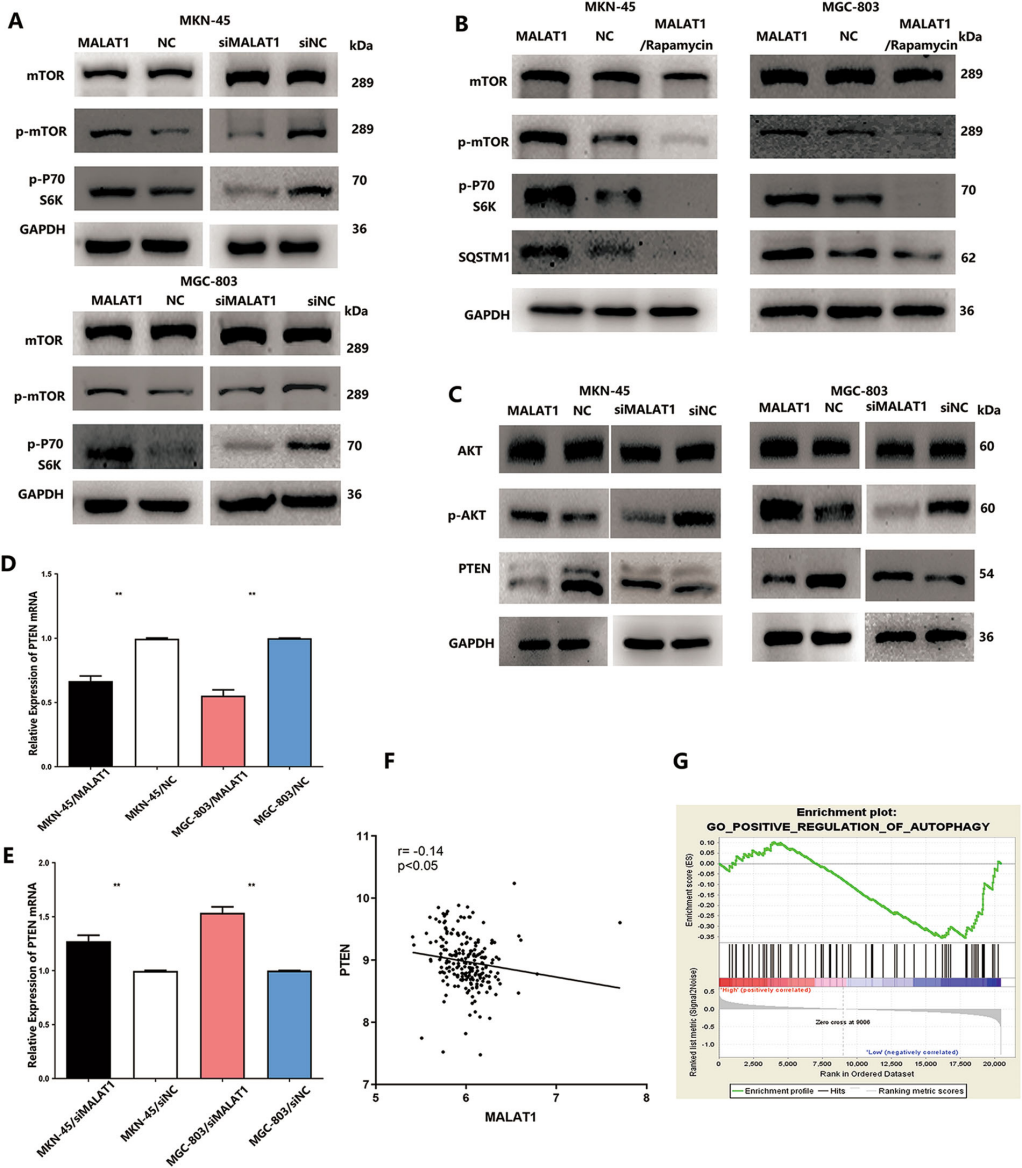
Increased MALAT1 regulated PTEN/AKT/mTOR pathway to inhibit autophagic flux. **A** The p-mTOR and p-p70S6K protein levels were increased in MKN-45 and MGC-803 cells transfected with MALAT1 overexpression vectors. Silencing MALAT1 resulted in p-mTOR and p-p70S6K level reduction; **B** The p-mTOR, p-p70S6K, and SQSTM1 protein levels were detected in MKN-45/MALAT1 and MGC-803/MALAT1 in presence of rapamycin; **C** The p-AKT and PTEN protein levels were detected in MKN-45 and MGC-803 cells transfected with MALAT1 overexpression vectors. Silencing MALAT1 resulted in PTEN overexpression and p-AKT downregulation; **D**, **E** The PTEN mRNA levels were detected in MKN-45/MALAT1 and MGC-803/MALAT1 cells. Silencing MALAT1 led to PTEN mRNA upregulation (MKN-45/MALAT1 vs MKN-45/NC: 0.66 ± 0.03 vs 1, MGC-803/MALAT1 vs MGC-803/NC: 0.53 ± 0.04 vs 1, *P* < 0.01; MKN-45/siMALAT1 vs MKN-45/siNC: 1.27 ± 0.04 vs 1 ± 0.01, MGC-803/siMALAT1 vs MGC-803/siNC: 1.53 ± 0.04 vs 1 ± 0.01, *P* < 0.01); **F** GEO dataset analysis showed a negative correlation between MALAT1 and PTEN. **G** GESA dataset analysis showed a negative correlation between MALAT1 and autophagy pathway (NES = −1.459, FDR *q*-value=0.06); Bars, S.D.; *P < 0.05; **P < 0.01; ***P < 0.001.

Activation of mTOR is crucial to inhibit autophagy flux, which led to substantial autophagosome–lysosome fusion inhibition and lysosome dysfunction so that autophagy degradation was impaired^17,18^. Hence, both phosphorylated-mTOR (p-mTOR) and its key substrate, phosphorylated-p70 S6 kinase (p-p70S6K), were detected to assess the MALAT1 effect on mTOR pathway activation. As shown in Fig. 2A, a significant p-mTOR and p-p70S6K level increase was observed in MKN-45 and MGC-803 cells transfected with MALAT1 overexpression vectors. Silencing MALAT1 resulted in p-mTOR and p-p70S6K level reduction, which indicated that MALAT1 could activate the mTOR pathway (Fig. 2A, *P* < 0.05). Autophagy flux impaired by MALAT1 in GC led to SQSTM1 accumulation and rapamycin was used to inhibit mTOR activation to better understand whether MALAT1 impaired autophagy degradation to elevate SQSTM1 accumulation via the mTOR pathway. Rapamycin could promote SQSTM1 reduction through silencing and reversing mTOR activation induced by MALAT1 in MKN-45 and MGC-803 cells (Fig. 2B, *P* < 0.05). Since the canonical PTEN/AKT pathway could regulate mTOR activity, phosphorylated AKT and PTEN expressions were also detected. MALAT1 overexpression could obviously downregulate PTEN protein level and upregulate phosphorylated AKT levels in MKN-45 and MGC-803 cells (Fig. 2C, *P* < 0.05). In addition, we found that MALAT1 not only inhibited PTEN protein level but also negatively regulated PTEN mRNA expression in MKN-45 and MGC-803 cells (Fig. 2D, E, *P* < 0.05). Analysis of the GSE dataset (GSE26942) also indicated a strong negative correlation between MALAT1 and PTEN mRNA (Fig. 2F, *P* < 0.05). Furthermore, GESA dataset analysis was also performed to suggest that autophagy was negatively associated with MALAT1 expression (Fig. 2G, NES =−1.459, FDR *q*-value = 0.06). Taken together, increased MALAT1 could negatively regulate PTEN expression to activate AKT/mTOR pathway, thus impairing autophagy flux and further elevating SQSTM1 accumulation in GC cells.

### MALAT1 inhibits PTEN expression at the post-transcriptional level

**Fig. 3:**
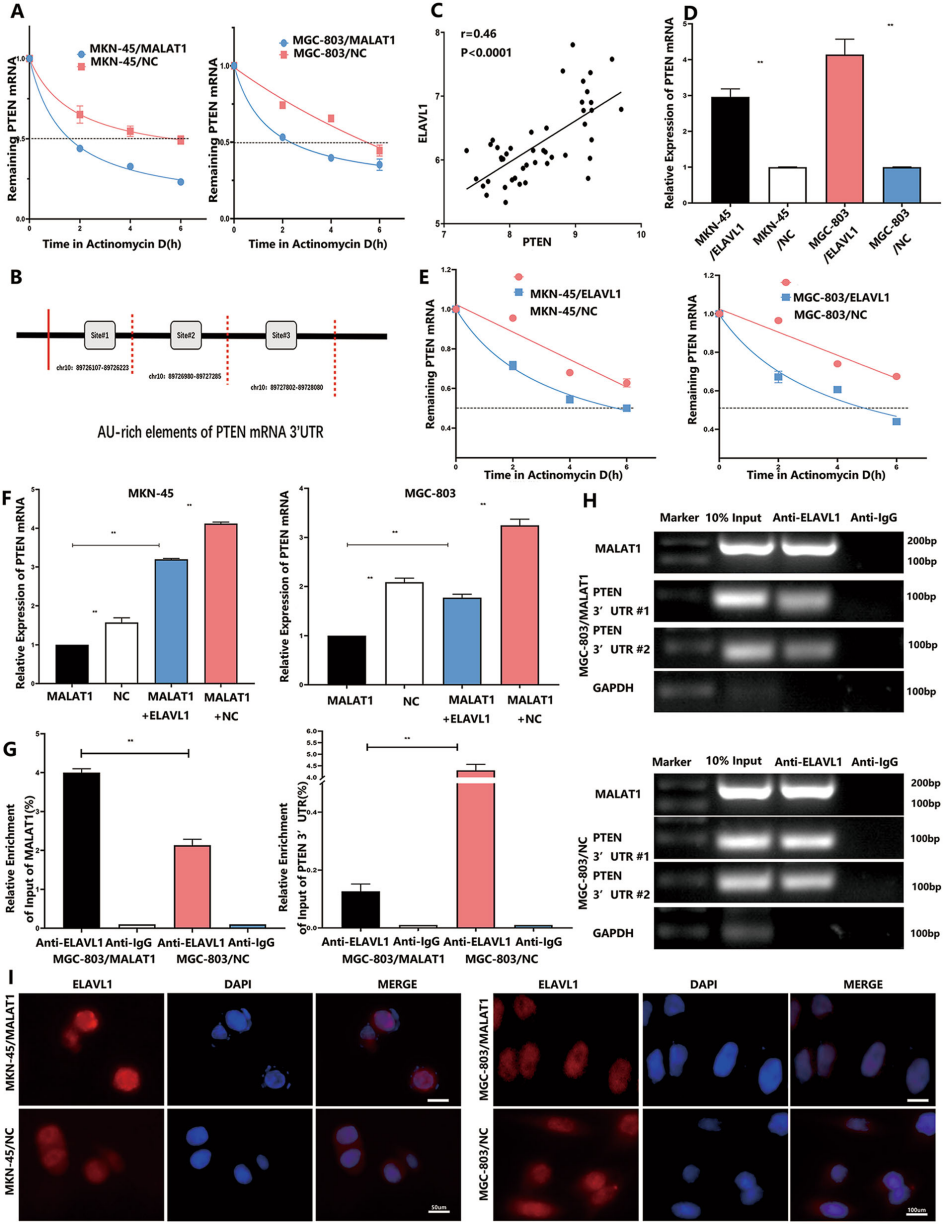
MALAT1 competitively interacted with ELAVL1 to destabilize PTEN mRNA. **A** PTEN mRNA expression in MKN-45 and MGC-803 transfected with MALAT1 overexpression vectors and the control after treatment with 5 μg/ml actinomycin D for 0, 2, 4, and 6 h. The PTEN transcript half-life was down-regulated by MALAT1; **B** Potential bindings sites of AU-rich elements on PTEN 3′-UTR; **C** GEO database analysis showed a positive correlation between ELAVL1 and PTEN; **D** ELAVL1 upregulation increase PTEN mRNA in MKN-45 and MGC-803 cells (MKN-45/ELAVL1 vs MKN-45/NC: 2.96 ± 0.18 vs 1 ± 0.01, MGC-803/ELAVL1 vs MGC-803/NC: 4.14 ± 0.35 vs 1 ± 0.01, *P* < 0.01); **E** PTEN mRNA expression in MKN-45 and MGC-803 transfected with ELAVL1 overexpression vectors and the control after treatment with 5 μg/ml actinomycin D for 0, 2, 4, and 6 h. The PTEN transcript half-life was upregulated by ELAVL1 (MKN-45/ELAVL1 vs MKN-45/MALAT1 + ELAVL1: 1 vs 3.2 ± 0.02, MGC-803/ELAVL1 vs MGC-803/MALAT1 + ELAVL1: 1 vs 1.80 ± 0.02, *P* < 0.05); **F** The remainder of ELAVL1-induced PTEN mRNA levels were abolished by increased MALAT1 in both MKN-45 and MGC-803 cells; **G**, **H** ELAVL1 captured more MALAT1 mRNA fractions than PTEN 3′-UTR enrichments under MALAT1 overexpression condition through performing RIP-PCR (MALAT1/Anti-ELAVL1 vs NC/Anti-ELAVL1: 4.0 ± 0.08 vs 2.13 ± 0.12 (MALAT1%), MALAT1/Anti-ELAVL1 vs NC/Anti-ELAVL1: 0.15 ± 0.04 vs 4.3 ± 0.21 (PTEN 3′-UTR%), *P* < 0.01); **I** MKN-45 and MGC-803 cells were transfected with MALAT1 overexpression vectors and the subcellular locations of HuR were determined by immunocytochemistry. Bars, SD; *P < 0.05; **P < 0.01; ***P < 0.001.

Although it has been demonstrated that MALAT1 could inhibit PTEN mRNA expression, the underlying mechanism has not been reported. Several studies have addressed the interaction between MALAT1 and the RNA binding protein (RBP) ELAVL1 to suppress target gene expression via modifying mRNA stability or mRNA initiation^19^. This inspired us to investigate whether MALAT1 regulates PTEN mRNA expression at the post-transcriptional level. The transcription inhibitor actinomycin D (Act D) was added in MKN-45 and MGC-803 cells transfected with or without MALAT1 plasmids for different times ranging from 0 to 6 h. The levels of remaining mRNAs were determined, and the PTEN mRNA half-lives decreased from 5.62 ± 0.21 to 1.54 ± 0.12 h and from 5.79 ± 0.18 to 2.47 ± 0.17 h (*P* < 0.01) in MKN-45 and MGC-803 cells, respectively, in response to increased MALAT1 (Fig. 3A, *P* < 0.01). AU-rich elements (AREs) usually exist in the 3′-UTR of mRNA, which could interact with RBPs to modulate mRNA stability. RBPmap database was used to analyse the ARE regions in PTEN 3′-UTR and predict the potential RBPs, which revealed that ARE regions were abundant in PTEN 3′-UTR and most possibly in ARE–ELAVL1 binding regions (Fig. 3B). Meanwhile, a significant positive correlation between ELAVL1 and PTEN mRNA expression was observed via analysing GEO datasets (GSE63048) (Fig. 3C, *P* < 0.001). Moreover, we found that ELAVL1 upregulation could increase PTEN mRNA expression in both MKN-45 and MGC-803 cells (Fig. 3D, *P* < 0.05). Similarly, after ELAVL1 overexpression, the PTEN mRNA half-lives increased from 6.16 ± 0.20 to 8.13 ± 0.28 h and from 5.29 ± 0.18 to 8.92 ± 0.35 h in MKN-45 and MGC-803 cells, respectively (Fig. 3E, *P* < 0.01). These results indicated that ELAVL1 could stabilize the PTEN mRNA. In addition, increased MALAT1 had no influence on ELAVL1 expression in GC cells (Supplementary Fig. 2A). Based on the current evidence on the opposite effects of MALAT1 and ELAVL1 on PTEN mRNA expression and the reported correlation between MALAT1 and ELAVL1, we assumed that increased MALAT1 could competitively interact with ELAVL1 to expose PTEN 3′-UTR such that PTEN mRNA destabilization was augmented. To determine the above assumption better, a rescue assay was carried out. As shown in Fig. 3F, ELAVL1-induced PTEN mRNA levels were significantly abolished by increased MALAT1 in both MKN-45 and MGC-803 cells (Fig. 3F, *P* < 0.05). Subsequently, RIP-PCR assay was performed to determine PTEN 3′-UTR enrichment bound by ELAVL1 with or without MALAT1 transfection in MGC-803 cells (Fig. 3G, H, *P* < 0.05) and MKN-45(Supplementary Fig. 2B, D, *P* < 0.05). These results showed that ELAVL1 could bind more MALAT1 mRNA fractions than PTEN 3′-UTR enrichments under MALAT1 overexpression condition. Additionally, we found that increased MALAT1 led to more ELAVL1 protein being distributed within the nucleus where MALAT1 was located (Fig. 3I), indicating that ELAVL1 nucleocytoplasmic shuttling was abrogated and resulted in PTEN mRNA destabilization. The combined data implied that MALAT1 could competitively interact with ELAVL1 to destabilize PTEN mRNA.

### Inhibition of autophagy promotes IL-6 secretion via accumulation of SQSTM1

**Fig. 4:**
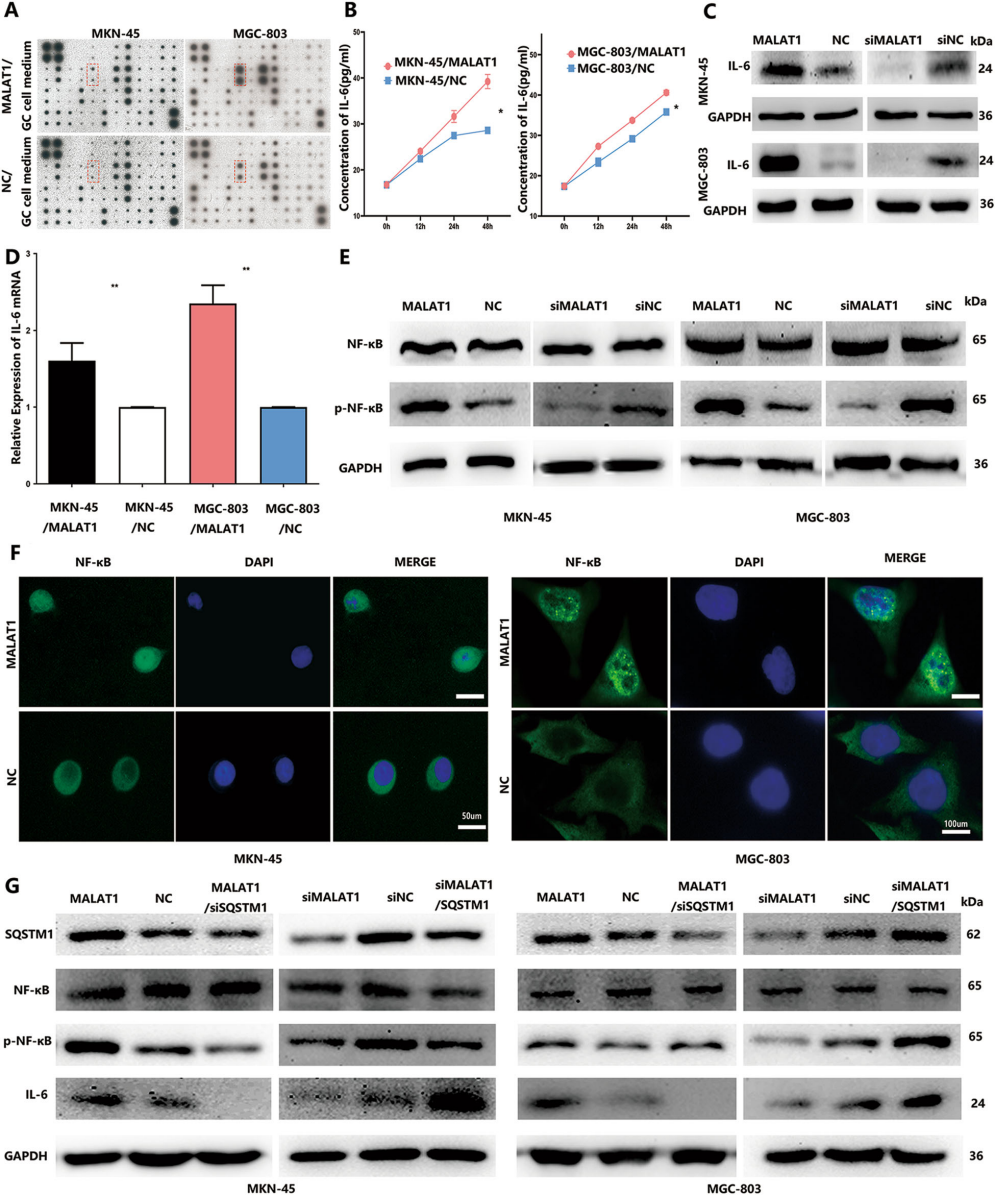
Blockage of autophagy induced IL-6 secretion via SQSTM1/NF-κB pathway activation. **A** Human cytokine antibody arrays were used to screen the difference of conditioned medium between GC cells transfected with MALAT1 overexpression vectors and NC vectors; **B** IL-6 protein expression level in the MKN-45/MALAT1, MGC-803/MALAT1, and compared groups was quantified 24 h after changing the culture medium as measured by ELISA (MKN-45/MALAT1 vs MKN-45/NC: 39.24 ± 1.24 vs 28.62 ± 0.17; MGC-803/MALAT1 vs MGC-803/NC: 40.6 ± 0.47 vs 35.79 ± 0.08, *P* < 0.05); **C**, **D** The mRNA and protein levels of p-IL-6 were detected in MKN-45 and MGC-803 cells transfected with MALAT1 overexpression vectors. Silencing MALAT1 resulted in IL-6 protein level downregulation (MKN-45/MALAT1 vs MKN-45/NC: 1.61 ± 0.2 vs 1 ± 0.07; MGC-803/MALAT1 vs MGC-803/NC: 2.35 ± 0.2 vs 1 ± 0.07, *P* < 0.01); **E** The NF-κB and p-NF-κB protein levels were increased in MKN-45 and MGC-803 cells transfected with MALAT1 overexpression vectors. Silencing MALAT1 resulted in p-NF-κB level reduction; **F** MKN-45 and MGC-803 cells were transfected with MALAT1 overexpression vectors, and the NF-κB subcellular locations were determined by immunofluorescence assay; **G** P-NF-κB and IL-6 expressions were abrogated in MKN-45/MALAT1 and MGC-803/MALAT1 cells with SQSTM1 siRNA treatment. Transfected SQSTM1 plasmid into MKN-45 and MGC-803 cells reversed NF-κB/IL-6 pathway inactivation caused by MALAT1 siRNAs. Three biological replicates were performed for in vitro assays. Bars, S.D.; *P < 0.05; **P < 0.01; ***P < 0.001.

Increasing evidence showed that autophagy flux inhibition aggravates the inflammatory response^20^. To investigate whether inhibition of autophagic flux inhibited by MALAT1 in GC cells could increase inflammatory cytokine release, a human cytokine antibody array was used to compare the conditioned media of MKN-45/MALAT1 and MGC-803/MALAT1 cells and those of MKN-45/NC and MGC-803/NC cells (Supplementary Table 1). The significant IL-6 increase was detected in cultured media (CM) of MKN-45/MALAT1 and MGC-803/MALAT1 cells (Fig. 4A), which was further confirmed by ELISA assay (Fig. 4B, *P* < 0.05). Next, we investigated the IL-6 expression in mRNA and protein level within GC cells transfected with MALAT1 plasmids, which demonstrated that increased MALAT1, could promote both IL-6 protein and mRNA expressions in MKN-45 and MGC-803 cells (Fig. 4C, D, *P* < 0.05). IL-6 expression is regulated by a wide range of transcription factors and NF-κB plays a crucial role. As expected, NF-κB activation and nuclear translocation were observed in MKN-45 and MGC-803 cells transfected with MALAT1 (Fig. 4E, F, *P* < 0.05). In addition, increased MALAT1 had no effect on SQSTM1 mRNA expression, which had been shown in the first part of the results. As mentioned above, increased MALAT1 impaired autophagic flux, resulting in elevated SQSTM1 accumulation within GC cells, with SQSTM1 being involved in both autophagy and inflammation response. Therefore, we assumed that MALAT1 might activate the NF-κB pathway to increase IL-6 expression via SQSTM1. SQSTM1 siRNA-treated MKN-45/MALAT1 and MGC-803/MALAT1 cells abrogated the enhanced phosphorylated-NF-κB and IL-6 expressions. Similarly, increased SQSTM1 via transfection with SQSTM1 plasmid could reverse the NF-κB/IL-6 inactivation pathway caused by MALAT1 siRNAs (Fig. 4G, *P* < 0.05). Taken together, increased MALAT1 could elevate SQSTM1 accumulation to activate NF-κB so that IL-6 expression could be increased.

### Impairment of autophagy in GC cells facilitates the transition from NFs to CAFs

**Fig. 5:**
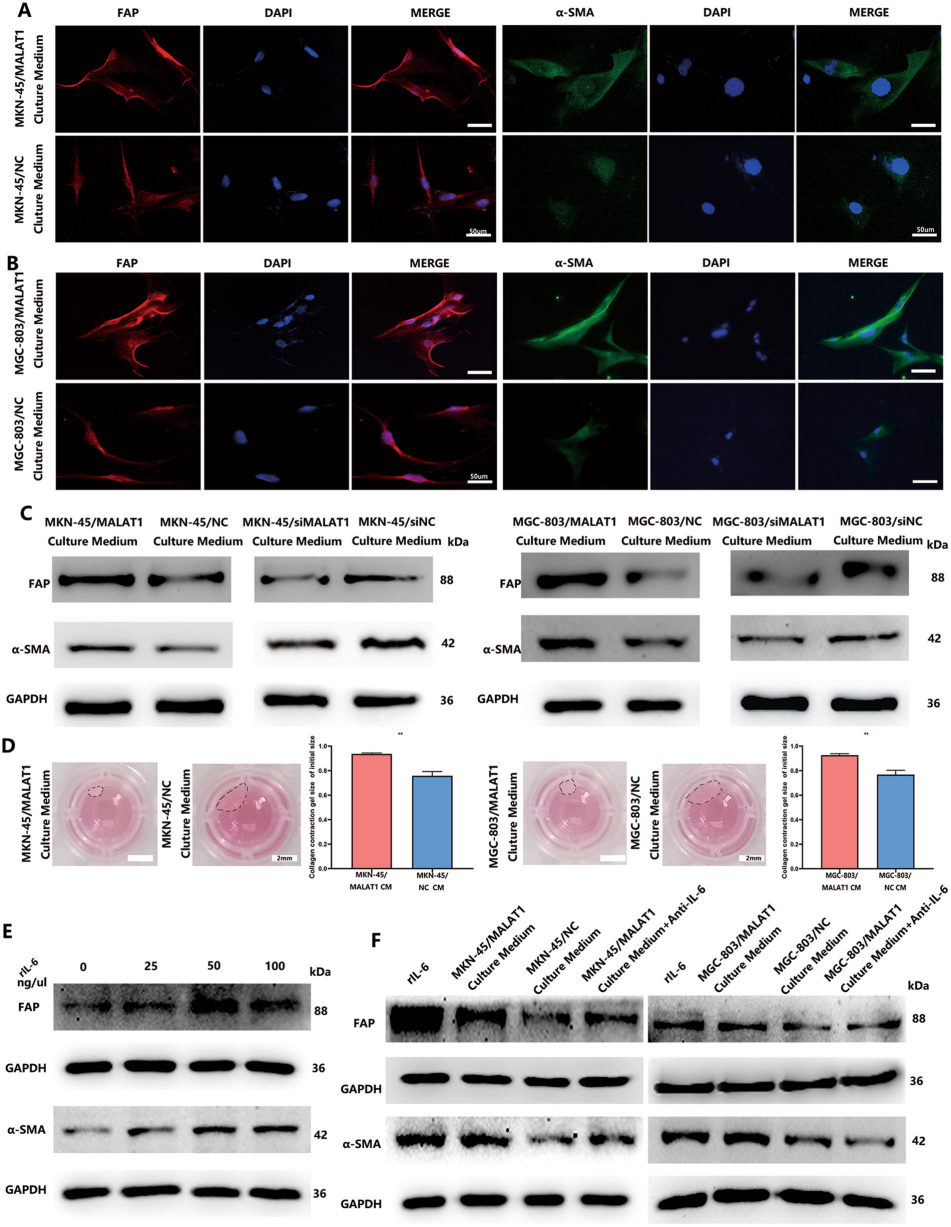
MALAT1-induced IL-6 from GC cells mediates NF–CAF transition. **A**–**C** Cultured medium collected from MKN-45/MALAT1 and MGC-803/MALAT1 cells induced NFs to acquire myofibroblast phenotype characterized by a-SMA and FAP expression as detected by Immunofluorescence and western blot assays; **D** NFs treated with cultured medium released by different GC cells or blank control were assessed for their ability to contract collagen; **E** The a-SMA and FAP protein levels were detected in NFs treated with different concentrations of reIL-6; **F** Protein levels of a-SMA and FAP in NFs co-cultured with cultured medium collected from MKN-45/MALAT1 and MGC-803/MALAT1 in the presence of IL-6 neutralizing antibody were analysed by western blot. Bars, S.D.; *P < 0.05; **P < 0.01; ***P < 0.001.

Existing evidence has revealed that inflammatory cytokines in TME can induce conversion of NFs to CAFs. Thus, autophagic flux impairment-induced inflammatory cytokine effect on NF activation was investigated. We found that the CM collected from MKN-45/MALAT1 and MGC-803/MALAT1 cells markedly induced NFs to acquire myofibroblast phenotype characterized by a-SMA and FAP expression (Fig. 5A–C). Furthermore, the fibroblast contraction abilities were markedly enhanced after treatment with CM derived from GC cells with increased MALAT1 (Fig. 5D, *P* < 0.05). To determine whether IL-6 was the dominant driver of this effect, NFs were treated with rIL-6, and the results demonstrated a dose-dependent FAP and a-SMA expression increase (Fig. 5E, *P* < 0.05). To better understand the paracrine effect of MKN-45/MALAT1- and MGC-803/MALAT1-secreted IL-6 on fibroblasts, the anti-IL-6 neutralizing antibody was used within rescue assay, which could weaken FAP and a-SMA expression in NFs treated with CM from GC cells with increased MALAT1 (Fig. 5F, *P* < 0.05). These results demonstrated that autophagy impairment-induced IL-6 from GC cells could activate NF to CAF conversion in a paracrine manner.

**Fig. 6:**
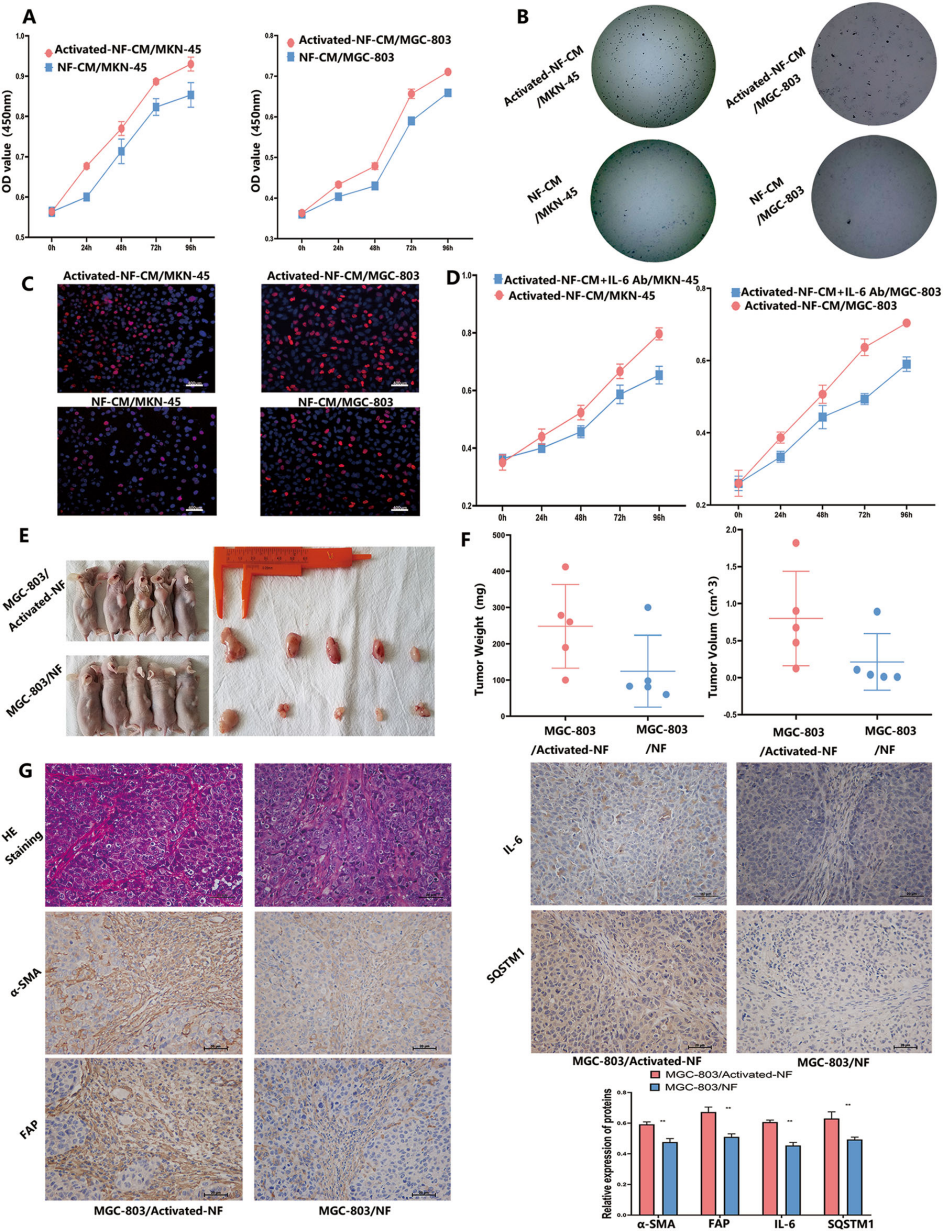
Activated fibroblasts promote GC progression. **A**–**C** Proliferation of MKN-45 and MGC-803 cells treated with activated-NF was determined by CCK8 (Activated-NF-CM/MKN-45 vs NF-CM/MKN-45: 0.93 ± 0.01 vs 0.85 ± 0.02; Activated-NF-CM/MGC-803 vs NF-CM/MGC-803: 0.74 ± 0.04 Vs 0.65 ± 0.01, *P* < 0.05), colony-formation (Activated-NF-CM/MKN-45 Vs NF-CM/MKN-45: 53.5 ± 11.8 Vs 28 ± 8.8; Activated-NF-CM/MGC-803 Vs NF-CM/MGC-803: 37.25 ± 8.8 Vs 7.25 ± 1.9, *P* < 0.05) and EdU (Activated-NF-CM/MKN-45 vs NF-CM/MKN-45: 35.3 ± 3.8 vs 12.6 ± 1.67; Activated-NF-CM/MGC-803 vs NF-CM/MGC-803: 33.3 ± 5.4 vs 18 ± 2.44, *P* < 0.05) assays; **D** Proliferation of MKN-45 and MGC-803 cells treated with activated-NF-CM and IL-6 blocking antibody was determined by CCK8 (Activated-NF-CM + IL-6 antibody/MKN-45 vs Activated-NF-CM/MKN-45: 0.79 ± 0.01 vs 0.65 ± 0.02; Activated-NF-CM + IL-6 antibody /MGC-803 vs NF-CM/MGC-803: 0.703 ± 0.01 Vs 0.59 ± 0.04, *P* < 0.01): **E** Photographs of tumours in nude mice derived from MGC-803 co-injected with activated-NFs and NFs; **F** MGC-803 mixed with activated-NFs generated tumours of larger volume and weight than those generated by MGC-803 mixed with NFs (MGC-803/activated-NFs vs MGC-803/NFs: 0.80 ± 0.5 vs 0.12 ± 0.34 cm^^3^; MGC-803/activated-NFs vs MGC-803/NFs: 248 ± 103 vs 124 ± 88 mg, *P* < 0.05); **G** FAP, a-SMA, SQSTM1 and IL-6 expressions were examined by IHC in tumours resulting from MGC-803/activated-NFs and MGC-803/NFs group. Bars, SD; *P < 0.05; **P < 0.01; ***P < 0.001.

To demonstrate the function of activated fibroblast converted from NFs (Activated-NFs), NFs treated with CM derived from GC cells with increased MALAT1 were prepared to perform proliferation assay and EdU dye assay. Then, MKN-45 and MGC-803 cells were incubated by CM collected from fibroblasts pre-co-cultured with MKN-45/MALAT1 and MGC-803/MALAT1 cells, respectively. As shown in Fig. 6A–C, GC cells exhibited proliferation and colony formation enhancement after treatment (Fig. 6A, C, *P* < 0.05). To further determine whether IL-6 from activated-NFs play a crucial role in promoting GC cells proliferation, and IL-6 blocking antibody was used to treat Activated-NF/MKN-45 and Activated-NF/MGC-803 group. Then we found that impairment of IL-6 with blocking antibody could significantly attenuate GC cells proliferation (Fig. 6D, *P* < 0.05). In addition, we further investigated whether activated-NFs could promote tumour growth in vivo. Co-injection of activated-NFs or NFs cells with MGC-803 was performed in nude mice. MGC-803 treated activated-NFs cells generated tumours with larger volume and weight than those generated by MGC-803 treated with NFs (Fig. 6E, F, *P* < 0.05). Furthermore, immunohistochemistry staining results showed that the FAP and a-SMA (CAF activation markers), SQSTM1 (autophagy marker) and IL-6 expressions were highly increased in MGC-803/activated-NF group (Fig. 6G, *P* < 0.01), which is consistent with in vitro experiment results.

### IL-6 derived from CAFs promote MALAT1 expression in GC cells

**Fig. 7:**
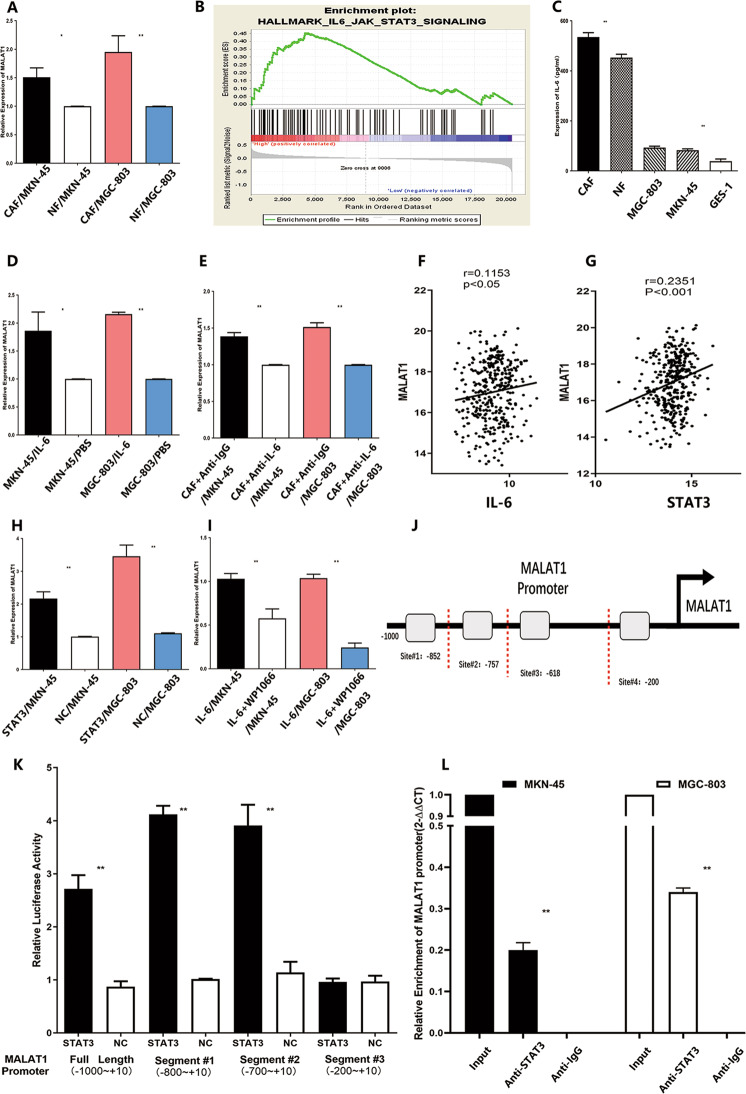
IL-6 derived from CAFs promote MALAT1 expression in GC cells. **A** Relative expression of MALAT1 was significantly higher in MKN-45 or MGC-803 co-cultured with CAFs than those co-cultured with NFs (CAF/MKN-45vs NF/MKN-45: 1.51 ± 0.13 vs 0.99 ± 0.01; CAF/MGC-803 vs NF/MGC-803: 1.95 ± 0.23 vs 0.99 ± 0.01, *P < 0.05, **P < 0.01); **B** GESA dataset analysis showed that IL-6/STAT3 pathway signalling had a positive association with MALAT1 expression(NES = 1.459, FDR *q*-value=0.26); **C** IL-6 was highly expressed in CAFs compared to NFs and GC cells(*P < 0.05, **P < 0.01); **D** The effect of reIL-6 (100 ng/mL) on MALAT1 expression in MKN-45 and MGC-803 were measured by qRT-PCR (MKN-45/IL-6 vs MKN-45/PBS: 1.86 ± 0.27 vs 1.03 ± 0.01: MGC-803/IL-6 vs MGC-803/PBS: 2.15 ± 0.03 vs 1.03 ± 0.01, *P < 0.05, **P < 0.01); **E** The effect of CAFs on MALAT1 expression in MKN-45 and MGC-803 cells was determined with the presence of IL-6 neutralizing antibody or IgG isotype control antibody (CAF + Anti-IgG/MKN-45 vs CAF + Anti-IL-6/MKN-45: 1.38 ± 0.04 vs. 1.02 ± 0.03; CAF + Anti-IgG/MGC-803 vs CAF + Anti-IL-6/MGC-803: 1.51 ± 0.04 vs 1.02 ± 0.03, **P < 0.01); **F**, **G** GEO dataset analysis suggested MALAT1 expression was positively associated with IL-6 and STAT3 expressions, respectively; **H** STAT3 upregulation promoted MALAT1 expression in MKN-45 and MGC-803 cells (STAT3/MKN-45 vs NC/MKN-45: 2.16 ± 0.16 vs 1: STAT3/MGC-803 vs NC/MGC-803: 3.46 ± 0.27 vs 1.1 ± 0.01, *P* < 0.01); **I** WP1066, a selective STAT3 inhibitor, could attenuate MALAT1 expression induced by recombinant IL-6 protein, which was measured by qRT-PCR (IL-6 + WP1066/MKN-45 vs IL-6+DMSO/MKN-45: 0.60 ± 0.07 vs 0.99 ± 0.03: IL-6 + WP1066/MGC-803 vs IL-6+DMSO/MGC-803: 0.24 ± 0.01 vs 0.99 ± 0.03, *P* < 0.01); **J** Potential STAT3 bindings sites on MALAT1 promoter; **K** Luciferase activity was measured after transfecting with MALAT1 promoter truncations, indicating that MALAT1 promoter site#3 contains binding sites (STAT3 vs NC: 3.91 ± 0.33 Vs 1.13 ± 0.17, *P* < 0.01); **L** Chip assay was performed to show that STAT3 could physically bind to MALAT1 promoter site#3 in MKN-45 and MGC-803 cells. Bars, S.D.; *P < 0.05; **P < 0.01; ***P < 0.001.

Increasing evidence showed that MALAT1 were aberrantly overexpressed and could act as an oncogene in GC^21^. The Cancer Genome Atlas (TCGA) data demonstrated that MALAT1 was highly expressed in GC (Supplementary Fig. 3A), and survival curve analysis with GEO dataset showed MALAT1 expression was negatively correlated with post-progression survival time of GC patients (Supplementary Fig. 3B). The cross-talk between CAFs and GC cells could aggravate the dysregulation of gene expression^22,23^. However, the mechanism of upregulation of MALAT1 within TME was rarely reported. Therefore, co-culture CAFs or NFs with GC cells were performed to determine whether CAFs could upregulate MALAT1 expression in GC cells via paracrine signalling. As shown in Fig. 7A, relative expression of MALAT1 was significantly higher in MKN-45 or MGC-803 co-cultured with CAFs group than that co-cultured with NFs group (Fig. 7A, *P* < 0.05), which means cytokine from CAFs might induce MALAT1 expression in GC. GESA dataset analysis was performed to suggest that IL-6/STAT3 pathway signalling was a positive association with MALAT1 expression (Fig. 7B, NES = 1.459, FDR *q*-value=0.26). Then the expression of IL-6 in CAFs, NFs and GC cells were determined by ELISA, which showed that IL-6 was dominantly overexpressed in CAFs (Fig. 7C, *P* < 0.01). Furthermore, higher expression of MALTA1 was detected in MKN-45 and MGC-803 cells treated with recombinant IL-6 protein (rIL-6) than that in MKN-45 and MGC-803 cells alone (Fig. 7D, *P* < 0.05). Blocking IL-6 activity with neutralizing IL-6 antibody of the co-culture system of CAFs and GC cells led to obvious impairment of MALAT1 expression (Fig. 7E, *P* < 0.05), indicating IL-6 derived from CAFs could promote MALAT1 expression in GC cells. GEO dataset (GSE60839) analysis showed overexpression of MALAT1 was significantly positive with the expression of IL-6 and STAT3 in GC samples (Fig. 7F, G, *P* < 0.05), suggesting STAT3 might be responsible for high expression of MALAT1 in GC.

Subsequently, upregulation of STAT3 expression via transfected STAT3 overexpression plasmid led to increasement of MALAT1 in both MKN-45 and MGC-803(Fig. 7H, *P* < 0.05). Furthermore, WP1066, a selective STAT3 inhibitor (inhibition of efficiency was shown in Supplementary Fig. 4C, D), could attenuate MALAT1 expression induced by recombinant IL-6 protein (Fig. 7I, *P* < 0.05), which suggested that IL-6 could increase MALAT1 expression via stimulating STAT3 activation. With help of the JASPAR database, we found that there are four most potential binding sites of STAT3 on MALAT1’s promoter accounting for the upregulation of MALAT1 in GC (Fig. 7J). To better understand whether STAT3 could interact with the MALAT1 promoter, a dual-luciferase reporter assay was carried out to measure luciferase activity after transfecting of truncations of the MALAT1 promoter. The results showed that site#3(-618bp~-200bp) of the MALAT1 promoter contains binding sites which mediated MALAT1 transcription activation induced by STAT3 (Fig. 7K, *P* < 0.01). Moreover, a CHIP assay was performed to show that STAT3 could physically bind to site#3(-618bp~-200bp) of the MALAT1 promoter in MKN-45 and MGC-803 cells (Fig. 7L, *P* < 0.01). Taken together, aberrant MALAT1 expression was partly attributed to IL-6 derived from CAFs via activation of the STAT3 pathway within GC TME. Additionally, we also found that overexpression of IL-6 was detected in MKN-45 and MGC-803 cells treated with rIL-6 (Supplementary Fig. 4E).

## Discussion

**Fig. 8:**
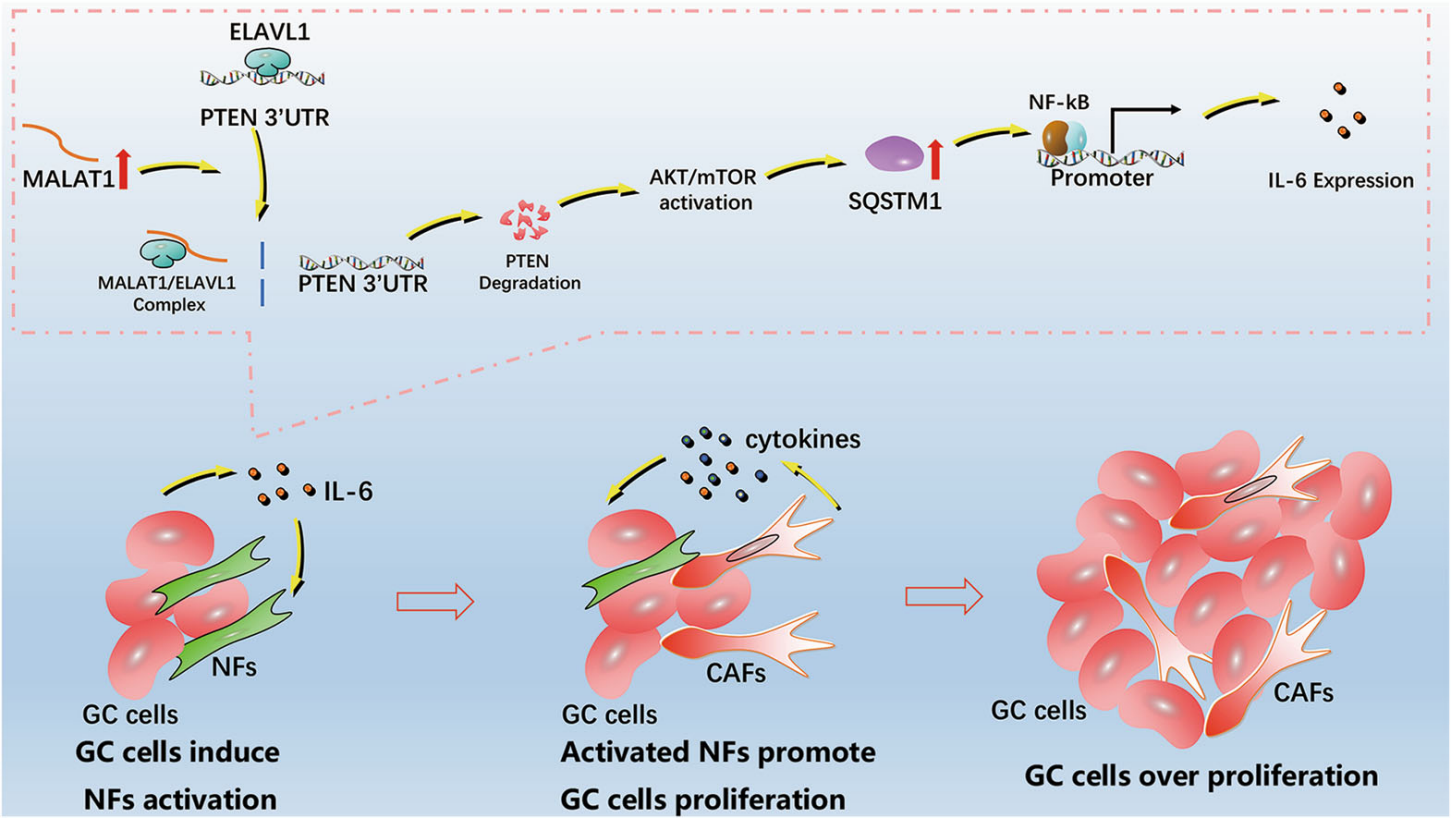
Schematic diagram summarizing the regulatory role of the MALAT1 in the interaction between GC cells and stromal cells. Increased MALAT1 in GC cells could impair autophagic flux to aggravate IL-6 secretion to activate converts NFs to CAFs via paracrine signalling, which resulted in GC cell progression. Increased MALAT1 could destabilize PTEN mRNA stability to activate AKT/mTOR pathway, which blocked autophagic flux leading to IL-6 overexpression induced by SQSTM1/NF-κB pathway. In addition, IL-6 secretion from GC cells stimulates NF conversion to CAFs.

In the present study, we showed, for the first time, that increased MALAT1 in GC cells could impair autophagic flux to aggravate IL-6 secretion to activate NF to CAF conversion via paracrine signalling, which resulted in GC cell progression. Increased MALAT1 could destabilize PTEN mRNA to activate AKT/mTOR pathway for blocking autophagic flux, leading to IL-6 overexpression induced by SQSTM1/NF-κB pathway, and the secreted IL-6 from GC cells stimulate NF to CAF conversion (Fig. 8). The interaction between GC and stromal cells could cause positive feedback to foster an inflammatory microenvironment and promote GC progression.

It has been widely reported that MALAT1 could function as versatile regulators by modulating transcription and post-transcriptional processes. Previously, we had found that MALAT1 could function as an oncogene to promote the proliferation of GC cells^24^. MALAT1 was overexpressed in GC and associated with TNM stages. Cancer cells play a vital role in tumour progression with the help of stromal cells within TME. However, the MALAT1 effect on the interaction between stromal and cancer cells have been rarely studied. Autophagy is a biological process involved with interaction between different types of cells through the production of inflammatory mediators^6,20^, which can regulate complex multicellular interactions within TME. Several studies have reported that MALAT1 could promote autophagy in various cancers, including retinoblastoma^13^, lung cancer^25^, and pancreatic cancer^11^. For GC, autophagy inhibition caused by increased MALAT1 has been rarely reported and investigated. For autophagy regulation, it is widely accepted that the mammalian target of rapamycin complex 1 (mTORC1) from the autophagy-inhibiting PI3k–Akt pathway^26^ and increased MALAT1 could activate PI3K–AKT pathway in numerous cancers including GC^27^. In this study, we demonstrated that increased MALAT1 could inhibit autophagy flux through activating AKT/mTOR pathway. Not only LC3-I to LC3-II protein conversion was increased along with MALAT1 augmentation in GC cells, SQSTM1 protein accumulation was also detected, suggesting LC3 protein conversion resulted from autophagy impairment rather than autophagy induction. In addition, rescue assays were performed to further confirm that increased MALAT1 could inhibit autophagy flux with 3-MA and BafA1 treatment. Besides the AKT/mTOR pathway being activated by increased MALAT1, expression of PTEN, the negative regulator of AKT/mTOR signalling, was also changed. Our study found that increased MALAT1 could destabilize PTEN mRNA to shorten its half-life in GC. AREs were rich in the PTEN 3′-UTR, to which RBP could bind to modulate mRNA stability. ELAVL1 is a ubiquitously expressed RBP that regulates many post-transcriptional steps including mRNA stability and translation. ELAVL1 has been reported to stabilize COX-2, β-catenin and BECN1 mRNA via binding to target AREs of 3′-UTR^28,29^. ELAVL1 not only could bind to 3′-UTR but also interact with lncRNA to form a functional complex. ELAVL1/MALAT1 complex was found to repress CD133 expression and suppress epithelial-mesenchymal transition in breast cancer^19^. However, whether ELAVL1 could bind to PTEN 3′-UTR regulating mRNA stability had not been reported, and whether MALAT1 could modulate PTEN mRNA expression via competitive interfering with the interaction between ELAVL1 and 3′-UTR was not investigated. In the present study, we found that MALAT1 could interact with ELAVL1 directly and restrain ELAVL1 in the nucleus away from the cytoplasm, where it could stabilize PTEN mRNA, as shown by RIP and IF assays. Based on collected evidence, we confirmed that increased MALAT1 could impair autophagy flux in GC via stimulating PTEN/AKT/mTOR signalling pathway. As a consequence of autophagy impairment caused by MALAT1, SQSTM1 accumulation was increased. Although expression of SQSTM1 was not investigated, several studies reported that SQSTM1 protein levels were more significantly upregulated in GC samples than in normal gastric mucosae^30,31^. SQSTM1 has been reported to be a significant activate factor in inflammatory responses^32,33^ through many signalling pathways including stimulating the NF-κB activation^34,35^. Therefore, we observed whether SQSTM1/NF-κB activation was responsible for IL-6 upregulation induced by increased MALAT1 in GC. From the results of rescue assays, we clearly found that SQSTM1 knockdown could reverse NF-κB activation and IL-6 upregulation caused by MALAT1, and restored SQSTM1 could reverse the NF-κB/IL-6 inhibition induced by silencing MALAT1 in GC cells.

CAFs secret inflammatory mediators to modulate components in TME and changes in TME can also regulate CAF function. We have shown previously that miR-149 can inhibit CAF activation via targeting IL-6 expression, which indicated that IL-6 has an important role in the CAF activation process^36^. In this study, we found that increased MALAT1 in GC cells results in IL-6 expression and secretion, and IL-6 augmentation activates NF to CAF conversion. The IL-6 effect on activating NFs was found in GC. IL-6 could also mediate the interaction between cancer cells and CAFs not only by supporting tumour cell growth but also by promoting fibroblast activation in oesophageal cancer^37^. Although IL-6 could stimulate NF to CAF conversion, the underlying molecular mechanisms were rarely known. Most studies attributed that IL-6 mediate the microRNA-dependent pathway to CAF activation^38–40^, which could not fully describe the underlying mechanisms. The mechanism of cytokines, like IL-6, on stimulating CAF activation should be further investigated. Chronic inflammation leads to NF activation and their conversion into CAFs, producing pro-tumorigenic cytokines, interacting with the cancer cells, and altering their gene expression profile, which results in cancer progression. In this study, activated CAFs induced by IL-6 could express α-SMA, acquire a highly contractile phenotype, and functionally, activated CAFs could facilitate GC cell proliferation, which resulted in co-evolution of CAFs with cancer cells. Additionally, MALAT1 has been reported to be aberrantly overexpressed in GC samples; however, the mechanism of upregulation of MALAT1 within TME was rarely reported. The interaction between CAFs and GC cells could aggravate the dysregulation of gene expression. We found that CAFs could upregulate MALAT1 expression in GC cells via paracrine signalling. Moreover, IL-6 derived from CAFs might be responsible for the high expression of MALAT1 in GC via promoting STAT3 binding to the MALAT1 promoter. In this way, the positive feedback loop contributed to positive feedback to foster an inflammatory microenvironment and promote GC progression.

In summary, our results indicate that MALAT1 could inhibit autophagic flux and instigate IL-6 via regulating PTEN/AKT/mTOR and SQSTM1/NF-κB pathways, which convert fibroblasts to CAFs to promote GC progression. (Fig. 8). However, the mechanism for CAF activation induced by IL-6 needs to be further investigated. Our study illustrated a new molecular mechanism underlying the interaction between cancer cells and fibroblasts, which may contribute to provide novel prevention and therapeutic strategies for GC.

## Materials and methods

### Cell lines

Human GC cell lines MKN-45, MGC-803, and GES-1 were purchased from the Shanghai Institute for Biological Sciences of the Chinese Academy of Sciences. They have been authenticated by an STR DNA profiling analysis and routinely examined for Mycoplasma contamination. GC cells were cultured in RPMI 1640 medium supplemented with 10% foetal bovine serum (FBS) and penicillin (100 μ/mL)/streptomycin (100 μg/mL) at 37 °C in 5% CO_2_ in air at saturation humidity.

### Isolation and culture of fibroblasts

CAFs and adjacent NFs were isolated from resected tissues from GC patients at the Department of Surgery, Ruijin hospital affiliated with Shanghai JiaoTong University, School of Medicine. The tissues were well cultured in Dulbecco’s modified Eagle’s medium (DMEM) with 10% FBS, 100 μ/mL penicillin and 100 ug/mL streptomycin. A homogeneous group of fibroblasts were developed after two weeks of culture, which were cultured >10 times so that the minimum number of clones could be selected. Identification test for CAFs and NFs were performed as described previously (Supplementary Fig. 4A, B). All patient samples were obtained with informed consent from Ruijin Hospital, Shanghai Jiao Tong University School of Medicine.

### RNA interference and plasmids

Small interfering RNAs (siRNAs) that specifically target human MALAT1 and SQSTM1 were purchased from Ribobio Technology (Guangzhou, China) and GenePharma (Shanghai, China), respectively. The siRNAs (100 nM siMALAT1, 100 nM siSQSTM1) were transfected into cells using the RNAi-MAX reagent (Life Technologies, CA, USA) according to the manufacturer’s instructions. The pcDNA-MALAT1 plasmid was kindly gifted by Prof. Huating Wang (The Chinese University of Hong Kong, China). Human ELAVL1 expression plasmids were purchased from Sangon Biotech (Shanghai, China). Plasmids (4 mg/ml) were transfected into cells using Lipofectamine 3000 (Life Technologies). Stably transfected cells (MGC-803/MALAT1, MGC-803/NC) were selected by using puromycin (1 mg/ml; InvivoGen). The RNA interference sequences are listed in Supplementary Table 1.

### Quantitative reverse transcription PCR (qRT-PCR)

Total RNA was extracted with TRIzol® reagent (Invitrogen, Austin, TX, USA), and real-time PCR analysis was conducted according to the manufacturer’s instructions (Life Technologies). The mRNA level was measured using the SYBR Green PCR Master Mix (Applied Biosystems, Waltham, MA, USA) and normalized to glyceraldehyde 3-phosphate dehydrogenase (GAPDH) mRNA level. The primer sequences used are listed in Supplementary Table 1.

### Western blot

Cells were lysed in RIPA buffer containing complete protease and phosphatase inhibitor cocktail (Sigma, USA). The protein concentration of the cell lysates was quantified by a BCA Protein Assay Kit (Pierce, Rockford). The same amount of protein samples was resolved onto 10% SDS-PAGE and then transferred to PVDF membranes. After blocking with 5% non-fat milk at 37 °C for 2 h, the membranes were incubated with the primary antibodies (1: 1000) diluted in TBST buffer overnight at 4 °C, followed by incubation with the HRP-conjugated secondary antibody for 2 h at room temperature. GAPDH antibody was used to verify equal protein loading. The protein band images were captured and analysed by a Tanon detection system with ECL reagent (Thermo) and the antigen-antibody reaction was visualized by enhanced chemiluminescence (ECL, Thermo, USA). The antibodies used in this study were obtained from Cell Signaling Technology.

### Transfection of mRFP-GFP-LC3 lentivirus vector

The mRFP-GFP-LC3 lentivirus vector was purchased from Genechem (Shanghai, China), which was transfected to GC cells according to the manual. Puromycin (1 μg/ml) was used to select stably expressing mRFP-GFP-LC3 cells. GC cells treated with different plasmids were fixed and analysed using fluorescence microscopy.

### Transmission electron microscopy (TEM)

GC cells were fixed in 2% glutaraldehyde containing 0.1 mol/l phosphate-buffered saline at 4 °C for 2 h, incubated in 1% osmium tetroxide containing 0.1 mol/l phosphate-buffered saline for 1.5 h at 4 °C, dehydrated in graded ethanol, saturated in graded ethanol, embedded, cut into ultrathin sections, stained with lead citrate, and finally viewed using Philip CM-120 TEM (Philips, Netherlands).

### RNA stability assay

Transcription inhibitor Actinomycin D (Sigma-Aldrich, USA) was added to the culture medium of GC cells transfected with different plasmids for 0, 2, 4, and 6 h. Individual total RNA was harvested for qRT-PCR analysis. The relative mRNA decay rate was measured and fit into an exponential curve.

### RNA immunoprecipitation-quantitative PCR (RIP-PCR)

RIP assays were performed by using the Magna RIP RNA-Binding Protein Immunoprecipitation Kit (Millipore, USA) according to the manufacturer’s instructions. Briefly, cells were lysed in lysis buffer and the cleared lysates were immunoprecipitated with the indicated anti-ELAVL1 and anti-IgG antibodies (Cell Signaling Technology). Immunoprecipitated and input RNA was isolated and reverse transcribed for qRT-PCR amplifications with PTEN 3′-UTR-specific primers. mRNA relative expression level was normalized to input mRNA expression. The primers used for amplification are listed in Supplementary Table 1.

### Enzyme-linked immunosorbent assay (ELISA)

The human angiogenesis array (Raybiotech, USA) was used to analyse the soluble mediators according to the manufacturer’s protocol. A human IL-6 ELISA kit (Raybiotech) was used to determine the concentration of human IL-6 in the medium of different treatments according to the manufacturer’s instructions.

### Immunofluorescence (IF)/Immunohistochemistry (IHC)

For IF assay, GC cells were fixed with 4% paraformaldehyde for 15 min at room temperature, permeabilized with 0.5% Triton *X*-100, and blocked with 5% BSA for 2 h before incubation with primary antibodies including anti-ELAV1, anti-DAPI, anti-NF-KB, anti-FAP (1: 500, Cell Signaling Technology), and anti-*α*-SMA (1: 500, Abcam, USA) overnight at 4 °C. After incubation with fluorescent secondary antibody for 2 h, images were acquired by fluorescence microscope.

### Collagen contraction assays

A total of 1 × 10^5^ NFs were suspended in 100 μl DMEM, which was mixed with 100 μl of collagen mix containing 68.75 μl DMEM and 31.25 μl Type 1 Rat tail collagen (Solarbio, China), and added to one well of a 96-well plate at 37 °C for 30 min. After incubation with media derived from different treatments for 24 h, the gels were photographed and the contractions were evaluated by using the Image J program.

### Cell-proliferation/EdU assay

Cells were seeded into 96-well plates (1.0×10^5^cells/well) and cell proliferation was documented every 24 h for 4 days. Cell proliferation was assessed in triplicates by using the Cell Counting Kit-8 (Dojindo, Kumamoto, Japan) following the manufacturer’s instructions. EdU assay was performed using Cell-Light EdU Apollo 567 In Vitro Imaging Kit (Ribobio, Guangzhou, China) according to the manufacturer’s instructions.

### Xenograft assay

All the experiments were performed in accordance with the official recommendations of the Chinese animal community. Four-week-old male BALB/C nude mice were purchased from the Institute of Zoology, Chinese Academy of Sciences of Shanghai. All nude mice were randomized allocated into two groups, in which NFs and MGC-803/MALAT1 or MGC-803/NC cells mixed at the ratio of 1:4 in 100 lL PBS were injected subcutaneously. During the experiment, the tumour volume was measured weekly using the formula *V* = (length × width^2^)/2.

### Statistical methods

Student’s *t*-test or one-way ANOVA were used for statistical analysis when appropriate. All statistical analyses were performed using SPSS 19.0 (SPSS Inc., Chicago, IL, USA). A two-tailed value of *P* < 0.05 was considered statistically significant. Gene set enrichment analysis (GSEA) was performed using GSEA v3.0 software.

## Supplementary information

Supplementary Fig. 1

Supplementary Fig. 2

Supplementary Fig. 3

Supplementary Fig. 4

Supplementary Figure legends

Supplementary Table1
