# Supplementary figures and images for: LncRNA MALAT1 promotes gastric cancer progression via inhibiting autophagic flux and inducing fibroblast activation
Source: Cell Death Dis. 2021 Apr 6;12(4):368. doi: 10.1038/s41419-021-03645-4 (PMC8024309; doi:10.1038/s41419-021-03645-4)

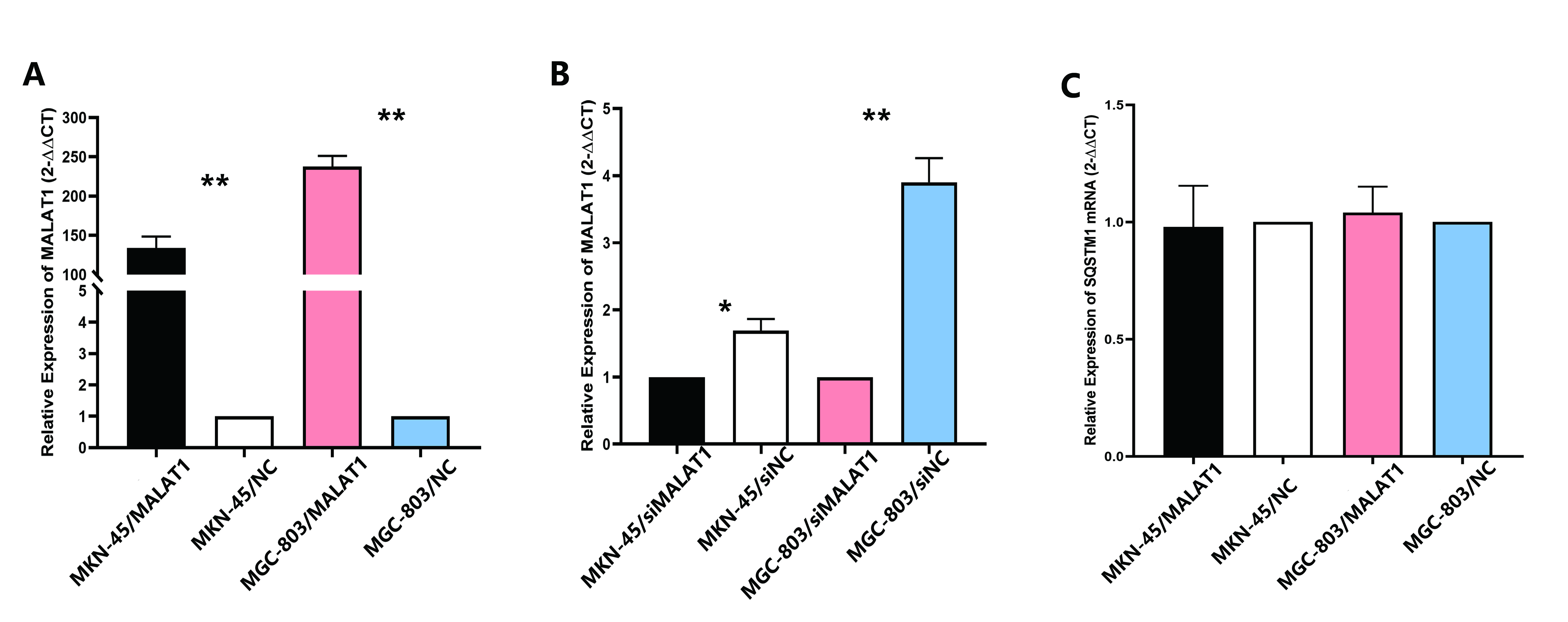

Supplement: Supplementary file 1 — Supplementary Fig. 1 [file 41419_2021_3645_MOESM1_ESM.jpg]

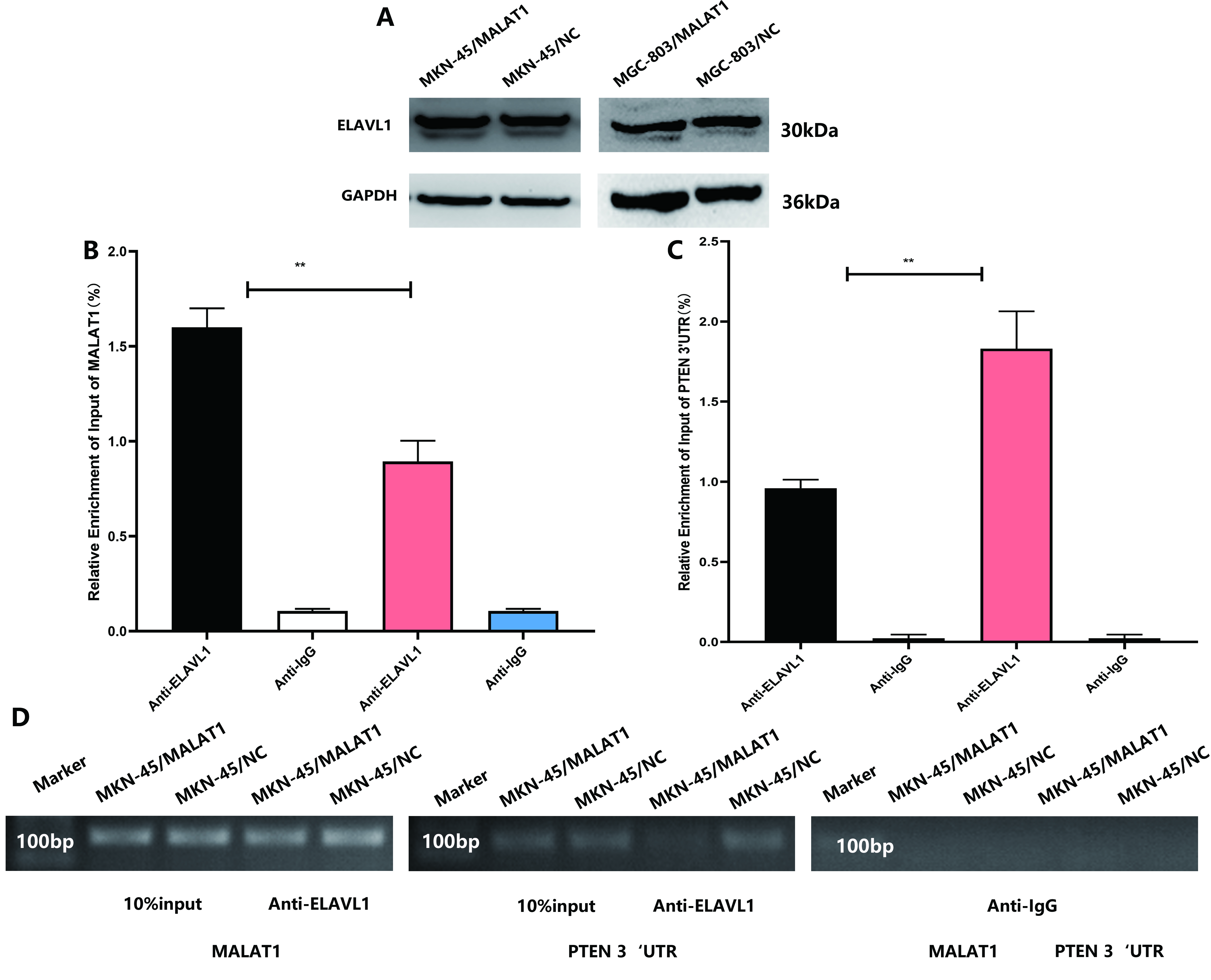

Supplement: Supplementary file 2 — Supplementary Fig. 2 [file 41419_2021_3645_MOESM2_ESM.jpg]

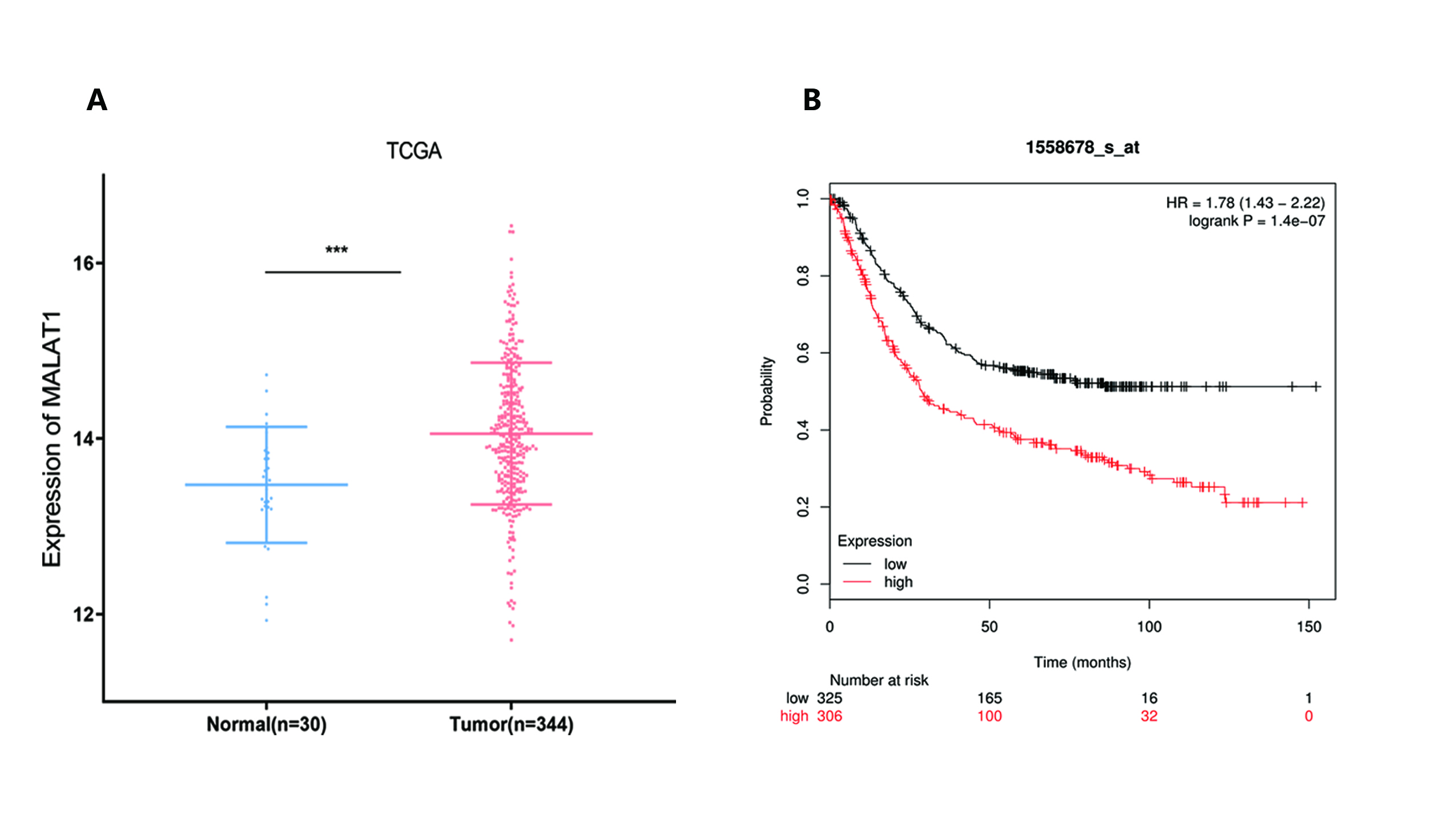

Supplement: Supplementary file 3 — Supplementary Fig. 3 [file 41419_2021_3645_MOESM3_ESM.jpg]

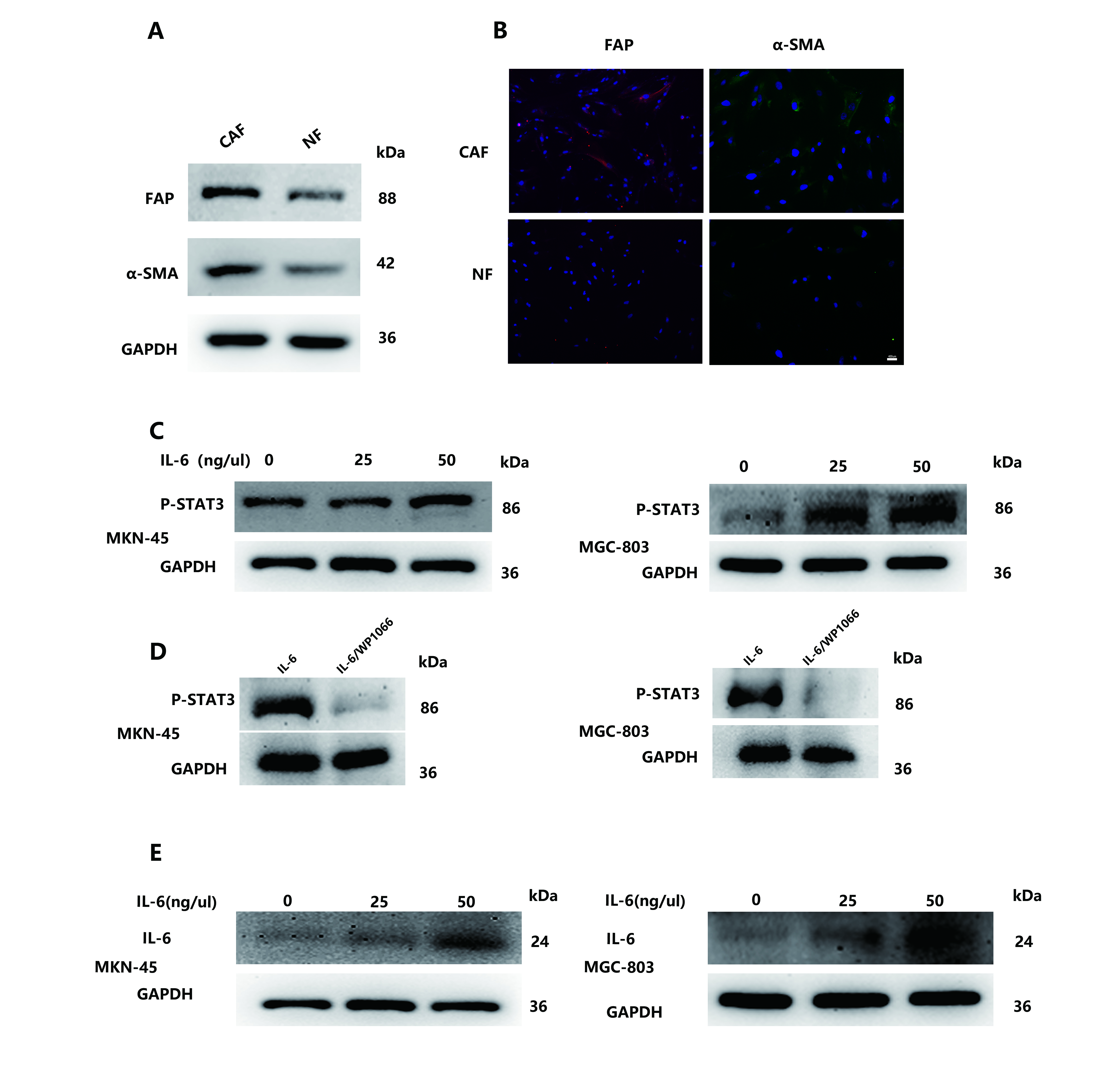

Supplement: Supplementary file 4 — Supplementary Fig. 4 [file 41419_2021_3645_MOESM4_ESM.jpg]
