# Supplementary material for: LncRNA MALAT1 promotes gastric cancer progression via inhibiting autophagic flux and inducing fibroblast activation
Source: Cell Death Dis. 2021 Apr 6;12(4):368. doi: 10.1038/s41419-021-03645-4 (PMC8024309; doi:10.1038/s41419-021-03645-4)
Supplement: Supplementary file 5 — Supplementary Figure legends [file 41419_2021_3645_MOESM5_ESM.docx]

**Supplementary Figure legends:**

**Supplementary Figure 1: A, B.** the expression levels of MALAT1 mRNA after transfecting MALAT1 plasmids and MALAT1 siRNAs, respectively; **C.** Increased MALAT1 had no effect on expression of SQSTM1 mRNA.

**Supplementary Figure 2: A.** Increased MALAT1 had no effect on expression of ELAVL1; **B-D.** ELAVL1 captured more MALAT1 mRNA fractions than PTEN 3′-UTR enrichments under MALAT1 overexpression condition through performing RIP-PCR.

**Supplementary Figure 3: A.** Analysis of TCGA data suggested MALAT1 was highly expressed in GC tissues (P=0.0057); **B.** Survival curve analysis with GEO dataset indicated MALAT1 expression was negatively correlated with post-progression survival time of GC patients.

**Supplementary Figure 4: A, B.** CAF phenotype characterized by a-SMA and FAP expression were determined by performing Immunofluorescence assay and western blot assay; **C.** recombinant IL-6 protein increased protein level of p-STAT3 in dose dependent manner; **D.** WP1066, a selective STAT3 inhibitor, inhibited activation of STAT3 induced by recombinant IL-6 protein; **E.** recombinant IL-6 protein increased protein level of IL-6 of GC cells in dose dependent manner.
