# Supplementary material for: LncRNA MALAT1 promotes gastric cancer progression via inhibiting autophagic flux and inducing fibroblast activation
Source: Cell Death Dis. 2021 Apr 6;12(4):368. doi: 10.1038/s41419-021-03645-4 (PMC8024309; doi:10.1038/s41419-021-03645-4)
Supplement: Supplementary file 6 — Supplementary Table1 [file 41419_2021_3645_MOESM6_ESM.pdf]

RiboTM h-MALAT1 Smart Silencer（MALAT1 siRNA）	GTTCAGAAGGTCTGAAGCTCdTdT
SQSTM1 siRNA	GUAAGCCUAGGUGUUGUCATT
Primers	
MALAT1 Forward	ATGCGAGTTGTTCTCCGTCT
MALAT1 Reverse	TATCTGCGGTTTCCTCAAGC
GAPDH Forward	CAATGACCCCTTCATTGACC
GAPDH Reverse	GACAAGCTTCCCGTTCTCAG
IL-6 Forward	CGGTCCAGTTGCCTTCTCCC
IL-6 Reverse	GAGTGGCTGTCTGTGTGGGG
PTEN Forward	GGTGGGTTATGGTCTTCAAAAGG
PTEN Reverse	TTTGAAGACCATAACCCACCAC
SQSTM1 Forward	GACTACGACTTGTGTAGCGTC
SQSTM1 Reverse	AGTGTCCGTGTTTCACCTTCC
MALAT1 promoter Forward	GCGTGGTAAGAGGCTCTCAAC
MALAT1 promoter Reverse	GACGTCAAGGCACTTTAAATGC
PTEN 3'UTR#1 Forward	AGAGTGACTACTGTGGAGTGGT
PTEN 3'UTR#1 Reverse	GGGAAGACAGAAAAAGAAAATGCCC
PTEN 3'UTR#2 Forward	AGTCTCTTGGTATGCATGGGGG
PTEN 3'UTR#2 Reverse	AGACTCAAGCCAAAGAAGAACAGAAAA

Relative expression levels of cytokines

Row	Column	Name	MKN-45/MALAT1 vs MKN-45/NC	MGC-803/MALAT1 vs MGC-803/NC
1,2,7,8	1,2,12	Pos(Averag	1	1
1,2	3,11	Neg	N/A	N/A
1,2	4	Neg	N/A	N/A
1,2	5	ENA-78	0.658490904	0.957587915
1,3	6	GCSF	0.151430231	0.974159137
1,4	7	GM-CSF	0.801138341	0.902738656
1,5	8	GRO a/b/g	0.976844179	0.9291891
1,6	9	GRO alpha	0.670418339	0.773311732
1,7	10	I-309	0.884879206	0.804223305
1,8	11	IL-1 alpha	0.990624647	1.061337388
1,9	12	IL-1 beta	0.80576893	1.706579569
3,4	1	IL-2	1.513528157	2.32369976
3,4	2	IL-3	1.083811904	1.005388013
3,4	3	IL-4	0.712833398	1.611402285
3,4	4	IL-5	1.133431759	1.49241915
3,4	5	IL-6	1.544433157	2.328245917
3,4	6	IL-7	0.51153105	2.24593409
3,4	7	IL-8	1.104263357	1.077188638
3,4	8	IL-10	0.958084828	0.971422309
3,4	9	IL-12 p40/p	1.005425917	0.64112196
3,4	10	IL-13	0.956465383	1.062744586
3,4	11	IL-15	1.54573651	0.546276747
3,4	12	IFN-gamma	0.930760757	0.649016859
5,6	1	MCP-1	1.168771748	1.104746998
5,6	2	MCP-2	1.381379767	1.288935334
5,6	3	MCP-3	1.58997778	1.3395838
5,6	4	M-CSF	1.124471925	0.907768318
5,6	5	MDC	1.031726566	0.911966393
5,6	6	MIG	0.821743057	1.623995885
5,6	7	MIP-1 delta	0.564604753	0.95604339
5,6	8	RANTES	0.972901827	0.958860792
5,6	9	SCF	1.368968913	0.426942374
5,6	10	SDF-1	0.768338127	0.831544413
5,6	11	TARC	1.21497394	0.709801682
5,6	12	TGF	0.975556647	1.040148306
7,8	1	TNF alpha	1.121881796	1.105808464
7,8	2	TNF beta	0.985943811	0.903030068
7,8	3	EGF	1.162921569	0.494631833
7,8	4	IGF-1	4.524516376	0.493218505
7,8	5	Angiogenin	2.553761185	0.565326722
7,8	6	OSM	0.744109865	0.933857189
7,8	7	TPO	1.253721203	2.090425766
7,8	8	VEGF-A	0.578197443	0.573527696
7,8	9	PDGF-BB	0.831623964	0.89394973
7,8	10	Leptin	0.850946662	1.285957545
